# Modified Primers for the Identification of Nonpathogenic *Fusarium oxysporum* Isolates That Have Biological Control Potential against Fusarium Wilt of Cucumber in Taiwan

**DOI:** 10.1371/journal.pone.0065093

**Published:** 2013-06-07

**Authors:** Chaojen Wang, Yisheng Lin, Yinghong Lin, Wenhsin Chung

**Affiliations:** 1 Department of Plant Pathology, National Chung Hsing University, Taichung, Taiwan; 2 Department of Biotechnology, Asia University, Wufeng, Taichung, Taiwan; Soonchunhyang University, Republic of Korea

## Abstract

Previous investigations demonstrated that *Fusarium oxysporum* (*Fo*), which is not pathogenic to cucumbers, could serve as a biological control agent for managing Fusarium wilt of cucumber caused by *Fo* f. sp. *cucumerinum* (*Foc*) in Taiwan. However, thus far it has not been possible to separate the populations of pathogenic *Fo* from the nonpathogenic isolates that have biological control potential through their morphological characteristics. Although these two populations can be distinguished from one another using a bioassay, the work is laborious and time-consuming. In this study, a fragment of the intergenic spacer (IGS) region of ribosomal DNA from an *Fo* biological control agent, Fo366, was PCR-amplified with published general primers, FIGS11/FIGS12 and sequenced. A new primer, NPIGS-R, which was designed based on the IGS sequence, was paired with the FIGS11 primer. These primers were then evaluated for their specificity to amplify DNA from nonpathogenic *Fo* isolates that have biological control potential. The results showed that the modified primer pair, FIGS11/NPIGS-R, amplified a 500-bp DNA fragment from five of seven nonpathogenic *Fo* isolates. These five *Fo* isolates delayed symptom development of cucumber Fusarium wilt in greenhouse bioassay tests. Seventy-seven *Fo* isolates were obtained from the soil and plant tissues and then subjected to amplification using the modified primer pair; six samples showed positive amplification. These six isolates did not cause symptoms on cucumber seedlings when grown in peat moss infested with the isolates and delayed disease development when the same plants were subsequently inoculated with a virulent isolate of *Foc*. Therefore, the modified primer pair may prove useful for the identification of *Fo* isolates that are nonpathogenic to cucumber which can potentially act as biocontrol agents for Fusarium wilt of cucumber.

## Introduction

Numerous formae speciales of *Fusarium oxysporum* (*Fo*) Schlechtend:Fr. are important pathogens that cause wilt diseases in many different host plants. This species is a ubiquitous soil-inhabiting fungus that is also a normal constituent of fungal communities in the rhizospheres of plants [1], [2], [3]. Historically, pathogenic *Fo* isolates showing high host specificity have been classified into more than 150 formae speciales based on plant species and cultivars they infect [4]. Among these formae speciales, *F. oxysporum* f. sp. *cucumerinum* (*Foc*) is one of the important pathogens that causes cucumber Fusarium wilt in most production areas, including North America, Europe and Asia [5]. Currently, several methods are being employed to control this disease, including breeding for resistance, fungicide application, crop rotation, soil amendments, and biological control. However, breeding for resistance and crop rotation practices are time-consuming, and the use of fungicides can be environmentally hazardous [6]. Soil amendments are less hazardous to the environment, but efficacy often depends on soil structure and pH [7]. Among biocontrol agents evaluated for the control of Fusarium wilt caused by *Fo* formae speciales, the use of nonpathogenic *Fo* isolates appears to hold much promise.

Nonpathogenic *Fo* isolates have been used for the control of Fusarium wilt caused by various *Fo* formae speciales [8]. A nonpathogenic *Fo* strain, Fo47, has been shown to be an effective biocontrol agent for managing Fusarium wilt in several vegetable and flower crops [9]. The introduction of nonpathogenic *Fo* into the stems of sweet potatoes and carnations [10], [11] resulted in the control of Fusarium wilt diseases in each respective host. In Taiwan, there are several reports of nonpathogenic *Fo* isolates found to be useful for the control of Fusarium wilt [12], [13]. Furthermore, Chen [12] reported that the *Fo* isolate Fo366 reduced the severity of cucumber Fusarium wilt caused by *Foc*. However, Fravel *et al*. [8] and Alabouvette *et al.* [14] showed that not all nonpathogenic *Fo* isolates are effective biocontrol agents. Screening nonpathogenic *Fo* isolates to assess the potential to serve as biocontrol agents has been difficult and time consuming. Thus, establishing a new method for rapid and reliable identification of nonpathogenic *Fo* isolates that have potential for use as biocontrol agents could be very beneficial.

Polymerase chain reaction (PCR) is a useful tool for the molecular characterization of fungi [15]. Many reports indicate that fungal species with similar morphology can be further classified based on PCR results [16], [17], [18], [19]. The intra-species diversity of several fungi, including formae speciales and races, has been further distinguished using the PCR approach [20], [21]. Recent studies showed that the intergenic spacer (IGS) region of ribosomal DNA (rDNA) is a source of phylogenetic markers in *Fo* [22], [23], [24], [25], [26] and that the region amplified with the general primers FIGS11/FIGS12 is suitable for the study of populations, including relationships among the *Fo* isolates [22], [27]. The objectives of this study were to identify polymorphisms in the IGS region of rDNA that differentiate nonpathogenic from pathogenic *Fo* isolates and to develop a method to assess the efficacy of nonpathogenic *Fo* isolates for use as potential biocontrol agents to manage Fusarium wilt of cucumber.

## Materials and Methods

### Fungal Isolates and Culture Conditions

A total of 145 *Fusarium* spp. isolates were included in this study. They were selected to represent the diversity among formae speciales and locations of origin in Taiwan (Table 1). One hundred and twenty two isolates represented 15 different formae speciales; of these isolates, six were *Foc* isolates, including the ATCC16416 type. Also included in the 122 isolates were seven *Foc* vegetative compatibility group (VCG) type strains (ATCC204373-379) and four VCG type strains (ATCC204369-372) of *Fo* f. sp. *radicis-cucumerinum* (*Forc*). The remaining 15 *Fo* isolates were nonpathogenic to cucumber (Fo276, Fo366, Fo95020, Fo95021, Fo95022, Fo95024, Fo95026) and tomato (AV-006, AV-007, AV-010, AV-011, AV-012, AV-013-1, AV-013-2, AV-014) (provided from AVRDC, unpublished data) from Taiwan, and eight isolates of seven other *Fusarium* spp. (*F. concentricum*, *F. decemcellulare*, *F. equiseti*, *F. moniliforme*, *F. proliferatum*, *F. solani* and *F. verticillioides*) were used in this study (Table 1). These 15 nonpathogenic *Fo* isolates were recovered from the soil or plant tissues by plating on quintozene peptone agar (PCNB) medium [28]. These nonpathogenic isolates were shown to be nonpathogenic to cucumber or tomato seedlings [5].

**Table 1:** Identification code, *Fusarium oxysporum* forma specialis or source of isolation of other fungal species, geographic origin, pathogenicity test and their results of PCR amplification for each isolate used in this study.

Isolate	F. oxysporumf. sp. or others^a^	GeographicOrigin	Pathogenicity^b^/host	PCR Primers^c^	
				FIGS11/FIGS12	FIGS11/NPIGSR
Foc100	*cucumerinum*	Nantou, Taiwan	+/cucumber	+	-
Foc106	*cucumerinum*	Pingtung, Taiwan	+/cucumber	+	-
Foc151	*cucumerinum*	Nantou, Taiwan	+/cucumber	+	-
Foc183	*cucumerinum*	Chiai, Taiwan	+/cucumber	+	-
Foc829	*cucumerinum*	Taichung, Taiwan	+/cucumber	+	-
ATCC 16416	*cucumerinum*	Florida, USA	+/cucumber	+	-
ATCC 204369	*radicis-cucumerinum*	USA	ND/cucumber	+	-
ATCC 204370	*radicis-cucumerinum*	USA	ND/cucumber	+	-
ATCC 204371	*radicis-cucumerinum*	USA	ND/cucumber	+	-
ATCC 204372	*radicis-cucumerinum*	USA	ND/cucumber	+	-
ATCC 204373	*cucumerinum*	USA	ND/cucumber	+	-
ATCC 204374	*cucumerinum*	USA	ND/cucumber	+	-
ATCC 204375	*cucumerinum*	USA	ND/cucumber	+	-
ATCC 204376	*cucumerinum*	USA	ND/cucumber	+	-
ATCC 204377	*cucumerinum*	USA	ND/cucumber	+	-
ATCC 204378	*cucumerinum*	USA	ND/cucumber	+	-
ATCC 204379	*cucumerinum*	USA	ND/cucumber	+	-
Fob08	*basilici*	Taichung,, Taiwan	+/basil	+	-
Fob09	*basilici*	Taichung,, Taiwan	+/basil	+	-
Fob10	*basilici*	Taichung,, Taiwan	+/basil	+	-
Foch 11-28	*chrysanthemi*	Changhua, Taiwan	+/garland chrysanthemum	+	-
Focb-21	*cubense*	Taiwan	+/Banana	+	-
Focb-24	*cubense*	Taiwan	+/Banana	+	-
Focb25	*cubense*	Taiwan	+/Banana	+	-
Focb-T14	*cubense*	Taitung, Taiwan	+/Banana	+	-
Focb-T34	*cubense*	Taitung, Taiwan	+/Banana	+	-
Focb-T35	*cubense*	Taitung, Taiwan	+/Banana	+	-
Focb-T36	*cubense*	Taitung, Taiwan	+/Banana	+	-
Focb-T38	*cubense*	Taitung, Taiwan	+/Banana	+	-
Focb-T44	*cubense*	Taitung, Taiwan	+/Banana	+	-
Focb-T105	*cubense*	Nantou, Taiwan	+/Banana	+	-
Focb-132	*cubense*	Chiyi, Taiwan	+/Banana	+	-
Focb-135	*cubense*	Nantou, Taiwan	+/Banana	+	-
Focb-136	*cubense*	Nantou, Taiwan	+/Banana	+	-
Focb-137	*cubense*	Pingtung, Taiwan	+/Banana	+	-
Focb-138	*cubense*	Pingtung, Taiwan	+/Banana	+	-
Focb-3-1	*cubense*	Pingtung, Taiwan	+/Banana	+	-
Focb-3-3	*cubense*	Pingtung, Taiwan	+/Banana	+	-
Focb-4-2	*cubense*	Kaohsiung, Taiwan	+/Banana	+	-
Focb-6-2	*cubense*	Hualien, Taiwan	+/Banana	+	-
Focb-7-7	*cubense*	Taitung, Taiwan	+/Banana	+	-
Focb-7-9	*cubense*	Chiyi, Taiwan	+/Banana	+	-
Focb-7-13	*cubense*	Nantou, Taiwan	+/Banana	+	-
Focb-TN3	*cubense*	Kaohsiung, Taiwan	+/Banana	+	-
ATCC 38741	*cubense*	Taiwan	+/Banana	+	-
ATCC 76243	*cubense*	SJ. Queensland, Australia	+/Banana	+	-
ATCC 76247	*cubense*	Honduras	+/Banana	+	-
ATCC 76257	*cubense*	Honduras	+/Banana	+	-
ATCC 76262	*cubense*	Taiwan	+/Banana	+	-
ATCC 96285	*cubense*	SE. Queensland, Australia	+/Banana	+	-
ATCC 96288	*cubense*	N. Queensland, Australia	+/Banana	+	-
ATCC 96289	*cubense*	SE. Queensland, Australia	+/Banana	+	-
ATCC 96290	*cubense*	SE. Queensland, Australia	+/Banana	+	-
Fog01	*gladioli*	Pintung, Taiwan	+/Gladiolus	+	-
Fog03	*gladioli*	Kaohsung, Taiwan	+/Gladiolus	+	-
Fog050	*gladioli*	Pintung, Taiwan	+/Gladiolus	+	-
Fog051	*gladioli*	Pintung, Taiwan	+/Gladiolus	+	-
Fog052	*gladioli*	Pintung, Taiwan	+/Gladiolus	+	-
Fog053	*gladioli*	Pintung, Taiwan	+/Gladiolus	+	-
Fola-2	*lactucae*	Yunlin, Taiwan	+/lettuce	+	-
Fola-18	*lactucae*	Yunlin, Taiwan	+/lettuce	+	-
Fola-11-13	*lactucae*	Yunlin, Taiwan	+/lettuce	+	-
Fola-32-14	*lactucae*	Yunlin, Taiwan	+/lettuce	+	-
Fola 103-7	*lactucae*	Yublin, Taiwan	+/lettuce	+	-
Fola-106-1	*lactucae*	Yunlin, Taiwan	+/lettuce	+	-
Fola-106-3	*lactucae*	Yunlin, Taiwan	+/lettuce	+	-
Fola-10	*lactucae*	Taoyuan, Taiwan	+/lettuce	+	-
Fola-40	*lactucae*	Taoyuan, Taiwan	+/lettuce	+	-
ATCC 76616	*lactucae*	CA, USA	+/lettuce	+	-
Foli G-16	*lilii*	Changhua, Taiwan	+/lily	+	-
Foli002	*lilii*	Nantou, Taiwan	+/lily	+	-
Foli016	*lilii*	Taichung, Taiwan	+/lily	+	-
Foli025	*lilii*	Taichung, Taiwan	+/lily	+	-
Foli044	*lilii*	Taichung, Taiwan	+/lily	+	-
Foli046	*lilii*	Taichung, Taiwan	+/lily	+	-
Foli156	*lilii*	Taichung, Taiwan	+/lily	+	-
Foli169	*lilii*	Taichung, Taiwan	+/lily	+	-
Folu114	*luffae*	Nantou, Taiwan	+/loofah	+	-
Folu638	*luffae*	Kaohsiung, Taiwan	+/loofah	+	-
FoluDS2	*luffae*	Tainan, Taiwan	+/loofah	+	-
FoluO1	*luffae*	Nantou, Taiwan	+/loofah	+	-
Foly11A Race1	*lycopersici*	Hualien, Taiwan	+/tomato	+	-
Foly34-1 Race2	*lycopersici*	Hualien, Taiwan	+/tomato	+	-
Foly146 Race2	*lycopersici*	Hualien, Taiwan	+/tomato	+	-
Foly195 Race1	*lycopersici*	Hualien, Taiwan	+/tomato	+	-
Fom2	*melonis*	Tainan, Taiwan	+/muskmelon	+	-
Fom3	*melonis*	Tainan, Taiwan	+/muskmelon	+	-
Fom4	*melonis*	Tainan, Taiwan	-/muskmelon	+	-
Fom5	*melonis*	Taichung, Taiwan	+/muskmelon	+	-
Fom6	*melonis*	Taichung, Taiwan	+/muskmelon	+	-
Fomo33	*momordicae*	Taichung, Taiwan	+/bitter gourd	+	-
Fomo34	*momordicae*	Taichung, Taiwan	+/bitter gourd	+	-
Fomo35	*momordicae*	Taichung, Taiwan	+/bitter gourd	+	-
Fomo36	*momordicae*	Taichung, Taiwan	+/bitter gourd	+	-
Fon-K0104	*niveum*	Tainan, Taiwan	+/watermelon	+	-
Fon-K0105	*niveum*	Tainan, Taiwan	+/watermelon	+	-
Fon-D0201	*niveum*	Changhua, Taiwan	+/watermelon	+	-
Fon-D0502	*niveum*	Changhua, Taiwan	+/watermelon	+	-
Fon-D0503	*niveum*	Changhua, Taiwan	+/watermelon	+	-
Fon-D0604	*niveum*	Changhua, Taiwan	+/watermelon	+	-
Fon-D0703	*niveum*	Changhua, Taiwan	+/watermelon	+	-
Fon-H0103	*niveum*	Miaoli, Taiwan	+/watermelon	+	-
Fon-P0101	*niveum*	Nantou, Taiwan	+/watermelon	+	-
Fon-P0401	*niveum*	Nantou, Taiwan	+/watermelon	+	-
ATCC 42006	*niveum*	Taiwan	+/watermelon	+	-
ATCC 44293	*niveum*	California, USA	+/watermelon	+	-
ATCC 64104	*niveum*	Taiwan	+/watermelon	+	-
Fop04	*phaseoli*	Nantou, Taiwan	+/snap bean	+	-
Fop05	*phaseoli*	Nantou, Taiwan	+/snap bean	+	-
Fop06	*phaseoli*	Nantou, Taiwan	+/snap bean	+	-
F54	*tracheiphilum*	Pingtung**,** Taiwan	+/asparagus bean	+	-
F55	*tracheiphilum*	Pingtung**,** Taiwan	+/asparagus bean	+	-
F67	*tracheiphilum*	Taichung, Taiwan	+/asparagus bean	+	-
F74	*tracheiphilum*	USA.	+/asparagus bean	+	-
F75	*tracheiphilum*	USA.	+/asparagus bean	+	-
F80	*tracheiphilum*	USA.	+/asparagus bean	+	-
F85	*tracheiphilum*	USA.	+/asparagus bean	+	-
F95	*tracheiphilum*	Pingtung**,** Taiwan	+/asparagus bean	+	-
F97	*tracheiphilum*	Pingtung**,** Taiwan	+/asparagus bean	+	-
F99	*tracheiphilum*	Pingtung**,** Taiwan	+/asparagus bean	+	-
F101	*tracheiphilum*	Pingtung**,** Taiwan	+/asparagus bean	+	-
Fot60	*tracheiphilum*	Pingtung**,** Taiwan	+/asparagus bean	+	-
Fo276	*F. oxysporum*	Hualien, Taiwan	-/cucumber	+	+
Fo366	*F. oxysporum*	Hualien, Taiwan	-/cucumber	+	+
Fo95020	*F. oxysporum*	Taichung, Taiwan	-/cucumber	+	-
Fo95021	*F. oxysporum*	Taichung, Taiwan	-/cucumber	+	-
Fo95022	*F. oxysporum*	Taichung, Taiwan	-/cucumber	+	+
Fo95024	*F. oxysporum*	Nantou, Taiwan	-/cucumber	+	+
Fo95026	*F. oxysporum*	Hualien, Taiwan	-/cucumber	+	+
AV-006	*F. oxysporum*	Kaohsiung, Taiwan	-/tomato	+	-
AV-007	*F. oxysporum*	Ilan, Taiwan	-/tomato	+	-
AV-010	*F. oxysporum*	Nantou, Taiwan	-/tomato	+	-
AV-011	*F. oxysporum*	Tainan, Taiwan	-/tomato	+	-
AV-012	*F. oxysporum*	Ilan, Taiwan	-/tomato	+	-
AV-013-1	*F. oxysporum*	Nantou, Taiwan	-/tomato	+	-
AV-013-2	*F. oxysporum*	Nantou, Taiwan	-/tomato	+	-
AV-014	*F. oxysporum*	Kaohsiung, Taiwan	-/tomato	+	-
SJ2a	*F. solani*	Chiayi, Taiwan	+/orchid	+	-
939229-3	*F. verticillioides*	Yunlin, Taiwan	+/orchid	+	-
STP-01	*F. monilion*	Taiwan	ND/corn feed	+	-
Fu3	*F. equiseti*	Taiwan	ND	+	-
Fu7	*F. decemcellulare*	Taiwan	ND	+	-
Fu11	*F. concentricum*	Taiwan	ND	+	-
YPES2	*F. proliferatum*	Chiayi, Taiwan	+/orchid	+	-
176-3	*F. proliferatum*	Yunlin, Taiwan	+/orchid	+	-

a Pathogenic strains of *Fusarium oxysporum* were isolated from soil, seed, or diseased host tissue. The other *F. oxysporum* strains were isolated from soil or healthy plant tissue.

b *F. oxysporum* isolates were tested for their pathogenicity using the root dip assay on their respective hosts, and the symbol “+” means positive for pathogenicity; “-” means no disease; “ND” means not tested.

c The symbol “+” means the PCR product of the expected size obtained; “-” means no PCR product of the expected size obtained.

For long-term storage of the cultures used in this study, single spore isolates were grown on PDA plates at 28°C for 5 days. Agar disks (0.5 cm in diameter) were cut from the colony margins and transferred into test tubes (12 cm in length, 1.5 cm in diameter) containing a soil-agar medium (1% WA plus 10% loamy sand soil, autoclaved for 30 min at 121°C, 15 lbs). The tube cultures were incubated at room temperature, with caps kept loosely, until the soil-agar medium was dry. Then, the caps were tightened, and the cultures were stored at room temperature.

### Fungal DNA Extraction, PCR and Analysis of the IGS Region

DNA extraction was conducted by the method of Saitoh *et al*. [29] with some modification. Ten- to fourteen-day-old mycelia from single spore isolates grown on potato dextrose agar (PDA) slants were transferred with an inoculation needle into a microtube containing 500 µl lysis buffer (200 mM Tris-HCl, 50 mM ethylenediaminetetraacetic acid, 200 mM NaCl, 1% n-lauroylsarcosine sodium salt, pH 8.0). The mycelia were dispersed in the buffer and incubated for 20 to 30 min at room temperature. The mixture was centrifuged at 18,000 *g* (Rotor: Nr. 12154, Sigma 3k20) for 10 min at 4°C, and then the supernatant (300 µl) was transferred into a new microtube. After the supernatant was mixed with 750 µl of ethanol by inverting the tube, the DNA was precipitated by centrifugation at 18,000 *g* for 4 min at 4°C. After one wash with 70% ethanol, the DNA pellet was air dried and dissolved in 50 µl TE buffer (pH 8.0). The PCR reactions were performed with genomic DNA from five selected *Fo* isolates using the primer set FIGS11 (5′ – GTAAGCCGTCCTTCGCCTCG –3′)/FIGS12 (5′ – GCAAAATTCAATAGTATGGC –3′) to amplify a part of the IGS region [30]. The five selected isolates include the known nonpathogenic biocontrol isolate Fo366 and four formae speciales isolates identified as *F. oxysporum* f. sp. *cucumerinum* (Foc100), *F. oxysporum* f. sp. *luffae* (Fol114), *F. oxysporum* f. sp. *phaseoli* (Fop04) and *F. oxysporum* f. sp. *tracheiphilum* (Fot60). Amplified DNA fragments were sequenced (Mission Biotech Co., Taiwan) and searched using the BLAST algorithm in GenBank from the National Center for Biotechnology Information (NCBI, Bethesda, MD).

### Primer Design and PCR Amplification

To design a primer pair that could differentiate nonpathogenic *Fo* isolates with biological control capability (Fo366) from pathogenic *Fo* isolates, the partial IGS nucleotide sequence from the *Fo* isolate Fo366 was submitted to GenBank under accession number AB683869 and used to compare with sequences from pathogenic *Fo* isolates. In this study, there were 20 formae speciales gene sequences selected from the NCBI GenBank database (http://www.ncbi.nlm.nih.gov/genbank/index.html) that were used to compare with the identical region from Fo366. The isolates from different *F. oxysporum* formae speciales and accession numbers are as follows: *apii* (AB106048), *asparagi* (AB373827), *cepae* (AB306845), *cubense* (AY527732), *gladioli* (AB383677), *glycines* (AB373826), *lactucae* (AB373825), *lagenariae* (AB306847), *lilii* (AB383690), *loti* (EU313466), *lycopersici* (AB373820), *matthiolae* (AB306843), *medicaginis* (EU313446), *melonis* (AB306848), *pisi* (EU313451), *radicis-lycopersici* (AB373823), *rapae* (AB306834), *raphani* (AB306841), *spinaciae* (AB306844) and *tulipae* (EU313443). The alignment software Clustal X 1.81 was used to analyze the unique region of Fo366 and the above mentioned formae speciales, and the identical regions were used to design a new primer. For PCR amplification, the 25-µl PCR mixture contained 1 µl fungal DNA, PCR Master Mix (1.25 U Taq DNA polymerase, reaction buffer, 1.75 mM MgCl_2_, 200 µM dNTP and enzyme stabilizer) (Genemark Technology Co., Ltd., Taiwan) and 0.2 µM of each primer (FIGS11/FIGS12). The PCR reaction was performed under the following temperature cycles: 95°C for 2 min, followed by 30 cycles of denaturing at 95°C for 30 sec, annealing at 58°C for 1 min, and polymerizing at 72°C for 45 sec, and a final extension at 72°C for 10 min. All the PCR reactions were conducted at three times to confirm reproducibility.

### Specificity, Sensitivity and Application of the Primer Pair

To assess the specificity of the primer pair FIGS11/NPIGS-R to detect nonpathogenic *Fo* isolates, genomic DNA from the following 137 *Fo* isolates and 8 *Fusarium* spp. isolates were used as template DNA for the PCR assay: 15 formae speciales, which included one isolate each of *chrysanthemi*; 3 isolates each of *basilici* and *phaseoli*; 4 isolates each of *luffae, lycopersici*, *momordicae* and *radicis-cucumerinum*; 5 isolates of *melonis*; 6 isolates of *gladioli*; 8 isolates of *lilii*; 10 isolates of *lactucae*; 12 isolates of *tracheiphilum*; 13 isolates each of *cucumerinum* and *niveum*; 32 isolates of *cubense*, 15 nonpathogenic isolates of *Fo* and 8 isolates of seven different *Fusarium* spp. (Table 1). Each 25-µl PCR mixture contained 1 µl *Fo* DNA, PCR Master Mix (Genemark Technology Co., Ltd., Taiwan) and 0.2 µM of each specific primer. PCR amplification was performed under the following temperature cycles: 95°C for 2 min, followed by 30 cycles of denaturing at 95°C for 30 sec, annealing at 58°C fo r 1 min, and polymerizing at 72°C for 45 sec, and then a final extension at 72°C for 10 min. PCR products were subjected to electrophoresis in 1.5% agarose gels.

To evaluate the sensitivity of the test, the quality of Fo276 DNA was quantified in GeneQuant 100 classic spectrophotometer (GE Healtcare), and diluted into several concentrations from 200 to 10^-3^ ng using 1 µl of each concentration in each treatment as template DNA in a 25-µl PCR reaction volume. The sensitivity experiments were replicated three times with independent dilutions. Isolate Fo276 was used instead of Fo366 as a nonpathogenic *Fo* type strain because it shared 100% similarity in nucleotide sequence identity with Fo366 and because isolate Fo366 started to show a decrease in its capacity to control Fusarium wilt over the course of this study (Wang, unpublished data).

To detect the fungal colonization of cucumber roots by nonpathogenic *Fo* isolates, three cucumber seeds (Showy Green, Known-You seed Co., Ltd, Taiwan) were sown into three 5 cm × 5 cm plastic tray cells containing infested peat moss (2×10^6^ conidia/g) for each nonpathogenic isolate. Conidia were produced on 2- to 3-wk-old PDA plate cultures at 28°C. Conidia were washed from the plates with sterile water, filtered through Miracloth (Calbiochem, San Diego, CA, USA), and quantified by counting in an improved Neubauer bright-line counting chamber (Marienfeld, Germany). One week after sowing, root tissues were collected from the seedlings, and total genomic DNA was extracted using the Plant Genomic DNA Purification Kit (Genemark Technology Co., Ltd, Taiwan) and used as template DNA.

For the detection of Fo276 in soil, a 1.5×10^4^ conidia/ml suspension was prepared from PDA plate cultures, as described above, and diluted in 10-fold increments to obtain a series of conidial concentrations. One milliliter of each concentration was added to microtubes containing one gram of autoclaved soil to establish various concentrations from 150,000 to 150 conidia/g soil. Total genomic DNA from each treatment was extracted using the Soil Genomic DNA Purification Kit (Genemark Technology Co., Ltd, Taiwan). All the PCR reactions were conducted three times to confirm reproducibility.

### Evaluation of Biocontrol Potential of Nonpathogenic *Fo* Isolates

Inoculum for infesting soil in pots with the pathogenic *Foc* isolate (Foc100) was produced on an oat/sand medium [31]. The propagation medium (200 g oats, 200 g sand, and 400 ml distilled water), contained in 1-L flasks, was autoclaved for 20 min on two consecutive days. Twenty ml of a 1×10^6^ spores/ml suspension collected from PDA plate cultures were aseptically pipetted into each culture flask, which was then incubated for 2-3 wk at room temperature to allow for colonization. Subsequently, the contents of the culture flasks were air dried for 1 wk and triturated into a fine powder using a blender (RT-04, Rong Tsong Precision Technology Co., Taiwan). Dry inoculum was mixed with nonsterilized (shown to be *Foc*-free) Taichung field soil to achieve an inoculum level of 10^3^ propagules/g of dry soil, as determined by 10-fold serial dilution plating on PCNB medium.

To evaluate the potential of nonpathogenic *Fo* isolates in reducing the severity of Fusarium wilt of cucumber, 10 susceptible plants (Showy Green, Known-You seed Co., Ltd, Taiwan) per isolate were grown in 5 cm × 5 cm cell plastic trays containing noninfested peat moss or peat moss infested with conidia from nonpathogenic isolates as described in the previous section of this paper. Trays were seeded and held in the greenhouse for 10-14 days, at which time the seedlings were transplanted into 12.5 cm diameter pots containing either noninfested or Foc100-infested field soil with 10^3^ propagules/g of dry soil. These plants were incubated for 8 wk in a greenhouse at 25-35°C and observed for symptom development. Disease severity was assessed weekly on a 0-4 scale in which “0” = healthy plants, “1” = plants with yellowing of the cotyledons and the first leaf, “2” =  stunted plants or yellowing of less than half of the leaves, “3”  =  plants with stem yellowing, vascular discoloration and wilting of more than half of the leaves, and “4” =  plants completely wilted or dead. The disease severity for each replicate of each treatment was calculated by the following formula: (ΣS_i_ × N_i_) × 100/(4 × N_t_), where Si is the severity ratings 0 to 4, N_i_ is the number of plants in each rating, and N_t_ is the total number of rated plants.

### Use of FIGS11/NPIGS-R to Identify *Fo* Isolates with Biocontrol Potential

Seventy-seven *Fo* isolates, 63 from soil and 14 from plant tissues, were collected at various locations across Taiwan and evaluated by PCR using the FIGS11/NPIGS-R primer set for amplification of the 500-bp IGS fragment. Isolates that tested positive for amplification were tested for pathogenicity to cucumber seedlings in a greenhouse root dip-inoculation test (5) and for their potential to suppress Fusarium wilt development using the method described in the preceding section.

### Phylogenetic Analysis of Nonpathogenic *Fo* Showing Efficacy on Control Cucumber Wilting Based on EF-1α Gene and IGS Region

Previous studies indicated that *F. oxysporum* might be an opportunistic pathogen of human [32], [33]. For realizing the relationship between nonpathogenic *Fo* with biocontrol activity and the pathogenic *Fo* of human and plant, two DNA regions of translation elongation factor (EF-1α) gene and intergenic spacer (IGS) were used to amplify and analyze following the method reported by O’Donell et al. [33]. The primers for amplifying EF-1α gene and IGS were EF-1H (5′ -ATGGGTAAGGAAGACAAGAC - 3′)/EF-2T (5′ -GGAAGTACCAGTGATCATGTT- 3′) and CNS1 (5′-GAGACAAGCATATGACTACTG - 3′)/CNL12 (5′ -CTGAACGCCTCTAAGTCAG - 3′), respectively [34]. The amplified sequences were aligned by CLUSTAL X 1.8 [35], and further visual alignments were done in SEQUENCE ALIGNMENT EDITOR (Se-Al) v.2.0 [36]. In this study, 11 and 18 isolates of the pathogenic *Fo* from human and plant respectively were used for analysis. Moreover, two isolates *F. foetens* were used as outgroups. The isolate number, species, host and accession no. in GenBank database of these added *Fo* isolates were showed in Table 2. Phylogenetic analysis of the aligned sequences was done by distanced methods. The distance matrix for the aligned sequences was calculated with the neighbor-joining (NJ) method [37]. Reliability of the inferred trees was estimated by 1,000 bootstrap resampling using the same program. Bootstrap [38] values were generated with 1,000 replicate heuristic searches to estimate support for clade stability of the consensus tree using the same program.

**Table 2:** The reference isolates used for phylogenetic analysis in this study.

NRRL no.	Host/substrate	Species/forma specialis	Accession no.	
			EF-1	IGS
22519	*Cucumis melo*	*F. oxysporum* f. sp. *melonis*	FJ985266	FJ985448
22553	*Raphanus sativus*	*F. oxysporum* f. sp. *raphani*	FJ985273	FJ985463
22554	*Chrysanthemum* sp.	*F. oxysporum* f. sp. *tracheiphilum*	FJ985274	FJ985464
25375	Human	*F. oxysporum*	AY527521	FJ985470
25378	Human	*F. oxysporum*	AY527428	AY527624
25387	Human	*F. oxysporum*	AY527527	FJ985471
25594	*Ipomoea batatas*	*F. oxysporum* f. sp. *batatas*	AY337717	FJ985478
26024	*Musa*	*F. oxysporum*	AY527535	AY527732
26203	*Solanum esculentum*	*F. oxysporum* f. sp. *lycopersici*	AF008501	FJ985487
26360	Human eye	*F. oxysporum*	AY527522	AY527719
26363	Human peritoneal fluid	*F. oxysporum*	AY527434	AY527630
26367	Human	*F. oxysporum*	AY527529	AY527726
26374	Human	*F. oxysporum*	AF008483	AY527714
26413	*Momordica charantia*	*F. oxysporum* f. sp. *momordicae*	FJ985291	FJ985498
26679	Human	*F. oxysporum*	AY527526	AY527723
28031	Human	*F. oxysporum*	AY527523	AY527720
28687	Human	*F. oxysporum*	AY527525	AY527722
32958	Human	*F. oxysporum*	AY527504	AY527700
36110	*Musa* ‘Cavendish’	*F. oxysporum* f. sp. *cubense*	FJ985327	FJ985560
36114	*Musa* ‘Pisang Manurung’	*F. oxysporum* f. sp. *cubense*	FJ985328	FJ985561
36276	*Pisum sativum*	*F. oxysporum* f. sp. *pisi*	FJ985341	FJ985574
36389	*Ipomoea batatas*	*F. oxysporum* f. sp. *batatas*	FJ985352	FJ985585
36464	*Solanum esculentum*	*F. oxysporum* f. sp. *lycopersici*	FJ985355	FJ985588
36472	*Cucumis melo*	*F. oxysporum* f. sp. *melonis*	FJ985357	FJ985590
37616	*Pisum sativum*	*F. oxysporum* f. sp. *pisi*	FJ985359	FJ985592
38289	*Ipomoea batatas*	*F. oxysporum* f. sp. *batatas*	FJ985368	FJ985601
38318	*Ocimum basilicum*	*F. oxysporum* f. sp. *basilici*	FJ985381	FJ985615
38552	*Citrullus lanatus*	*F. oxysporum* f. sp. *niveum*	FJ985410	FJ985645
38591	*Cucumis sativus*	*F. oxysporum* f. sp. *cucumerinum*	FJ985379	FJ985613
31852	*Begonia elatior*	*F. foetens* (outgroup)	HM057337	HM057282
38302	*Pinus radiata* seedling	*F. foetens* (outgroup)	GU170559	GU170581

### Statistical Analyses

Data were analyzed with the software SPSS 10.0 for Windows® (LEAD Technologies, Inc., Charlotte, NC, USA). ANOVA was performed, and the Duncan post-hoc test was conducted to assess the differences among the treatments within each week at p = 0.05.

## Results

### Primer Design

**Figure 1:**
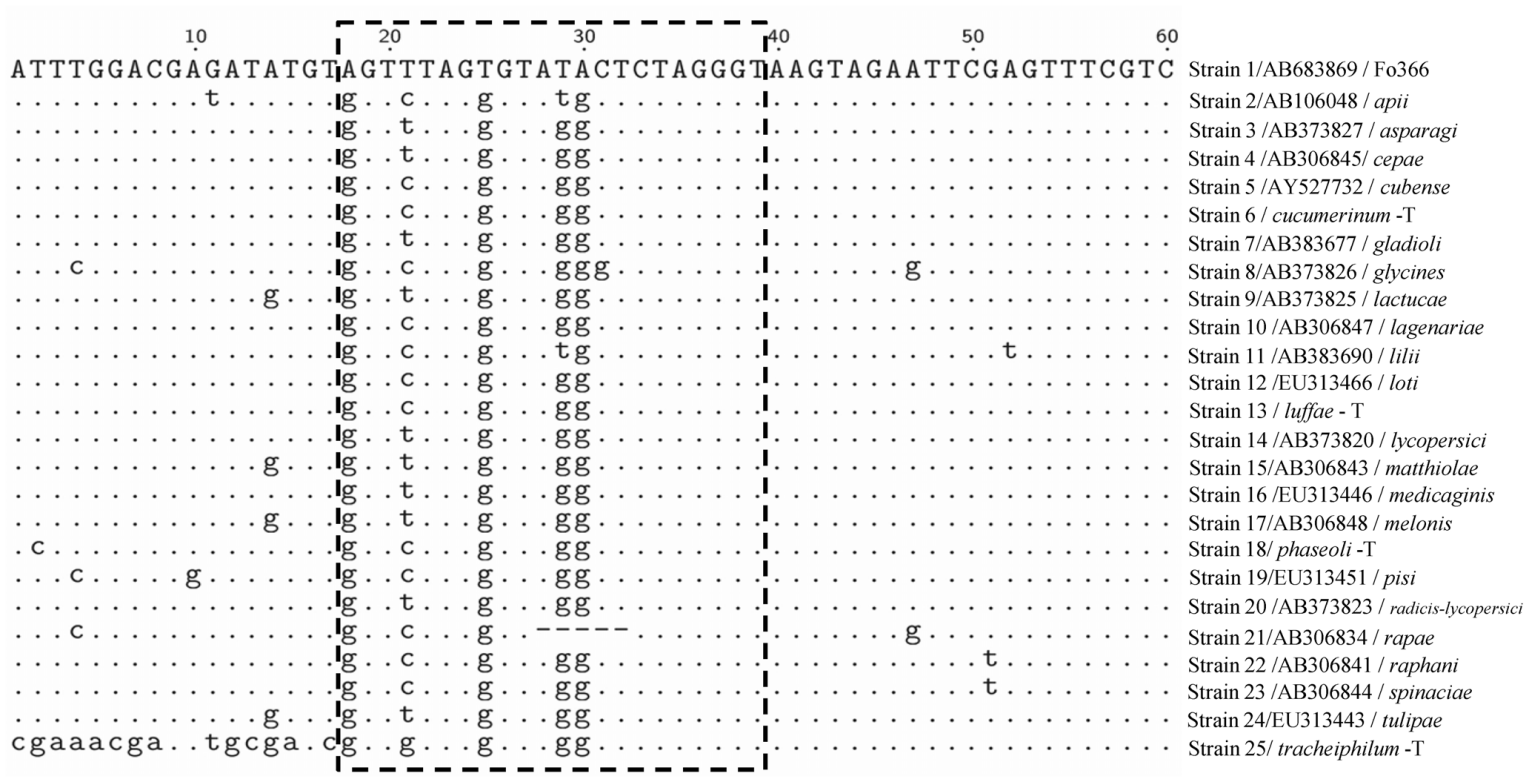
Nucleotide variation of nonpathogenic *Fo* isolates (Fo366 ) and other *Fo* formae speciales. Nucleotide sequence alignment of the rDNA repeats encoding a part of the intergenic spacer region (IGS) for strains of nonpathogenic *Fusarium oxysporum* (strain 1: Fo366) and pathogenic *F. oxysporum* of 24 formae speciales strains (strains 2-25 represented *apii, asparagi, cepae, cubense, cucumerinum-*T, *gladioli, glycines, lactucae, lagenariae, lilii, loti, luffae-*T, *lycopersici, matthiolae, medicaginis, melonis, phaseoli-*T, *pisi, radicis-lycopersici, rapae, raphani, spinaciae, tulipae* and *tracheiphilum-*T, respectively. The notation -T stand for the strains that were isolated from samples collected from Taiwan). The nucleotide bases in the Fo366 sequences different from the other 24 *formae speciles* are indicated below the sequences. Lowercase letters indicate the nucleotide bases that differ between the pathogenic and the (Fo366) nonpathogenic strains. The dashes indicate base gaps. The dashed line region represented the sequence of specific primer, NPIGS-R.

Amplified fragments of nonpathogenic *Fo* isolate Fo366 (strain 1) and four pathogenic *Fo* isolates (strains 6, 13, 18 and 25) were purified, sequenced and then compared for nucleotide variation with 20 other *Fo* formae speciales using Clustal X 1.81 alignment software (Fig 1). The specific primer NPIGS-R (5' - ACCCTAGAGTATACACTAAACT - 3') was designed according to the polymorphisms found in the IGS DNA sequence of Fo366 when compared with the formae speciales isolates (Fig 1).

### Specificity of the Modified Primer Pair

**Figure 2:**
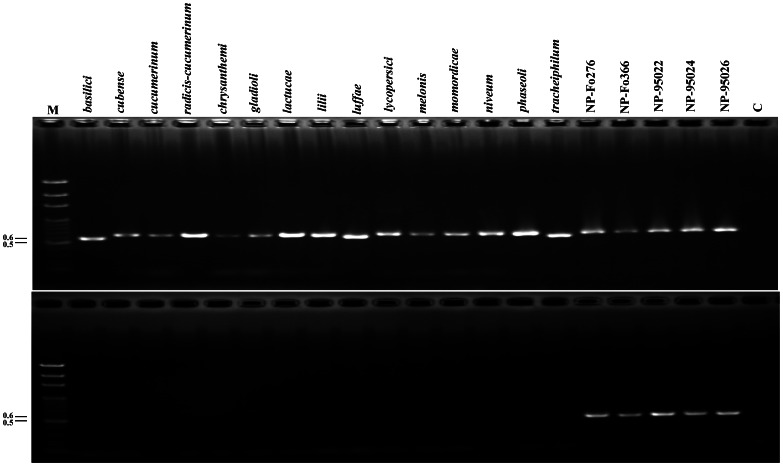
Specificity of the modified primer pair. Agarose gels showing the amplification products from polymerase chain reaction (PCR) using genomic DNA from isolates of 15 *formae speciales*, including *basilici*, *chrysanthemi*, *cubense*, *cucumerinum*, *radicis-cucumerinum*, *gladioli*, *lactucae*, *lilii*, *luffae*, *lycopersici*, *melonis*, *momordicae*, *niveum*, *phaseoli* and *tracheiphilum*, and five nonpathogenic strains of *Fusarium oxysporum* (*Fo*). (A) 550 to 650 bp DNA products of different *formae speciles* and nonpathogenic *Fo* isolates amplified by FIGS11/FIGS12. (B) 500-bp DNA product of five nonpathogenic *Fo* isolates amplified by new primer NPIGS-R and FIGS11. The numbers on the left are the molecular weights (Kb) of the Gen-100 bp DNA ladder (GeneMark) (lane M).

The newly designed primer NPIGS-R combined with FIGS11 was used to assess the amplification of 122 formae speciales isolates, eight different *Fusarium* spp. isolates and 15 nonpathogenic *Fo* isolates. No fragment or expected size was amplified from the 122 pathogenic *Fo* and 8 different *Fusarium* spp. isolates (Table 1). A 500-bp fragment was amplified from the *Fo* isolates Fo95022, Fo95024, Fo95026, Fo276 and Fo366, but no PCR product was amplified from other *Fo* isolates (Table 1). The PCR-amplified fragments of the *Fo* isolates Fo95022, Fo95024, Fo95026, and Fo276 were sequenced, and the nucleotide sequences showed 100% identity with Fo366. In contrast, all isolates, both pathogenic, nonpathogenic and different *Fusarium* spp., had a 550 to 700-bp fragment amplified using the primer pair FIGS11/FIGS12, thereby confirming the quality of the genomic DNA and the species of *F. oxysporum*. The electrophoresis picture of PCR reactions of partial *F. oxysporum* isolates was showed in Fig 2.

### Sensitivity and Application of the Newly Designed Primer

**Figure 3:**
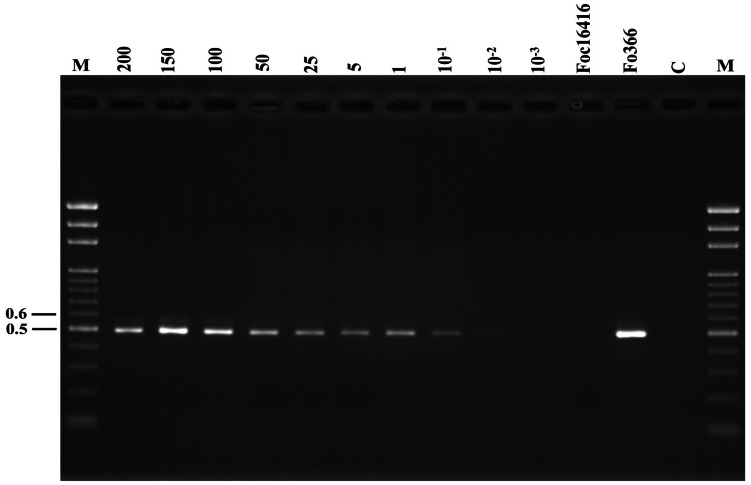
Sensitivity of the newly designed primer. Agarose gel showing the sensitivity of polymerase chain reaction (PCR) using the genomic DNA of a nonpathogenic strain of *Fusarium oxysporum* and the primer pair FIGS11/NPIGS-R: Amplification of a decreasing amount of the nonpathogenic isolate Fo276 DNA ranging from 200 to 10^-3^ ng. The numbers on the left correspond to the molecular weight (kb) of the Gen-100 ladder (lane M). Lanes Foc16416 and Fo366, the amplification controls for the pathogenic isolate of *F. oxysporum* f. sp. *cucumerinum* (Foc100) and nonpathogenic isolate Fo366 DNA, respectively. Lane C, control reaction with no template DNA.

**Figure 4:**
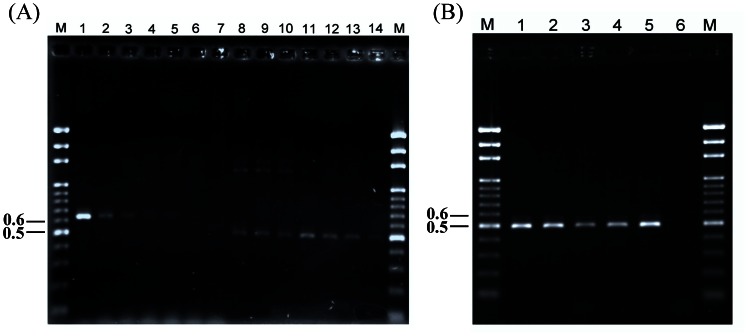
Application of the newly designed primer. The detection sensitivity of the primer sets FIGS11/FIGS12 and FIG11/NPIGS-R amplified DNA fragments of the nonpathogenic *Fusarium oxysporum* strain Fo276 in plant tissues (A) and soil particles (B). (A): The total genomic DNA of plant root tissues was diluted into different fold dilutions (1, 10, 20, 40, 80, 100 and 200) (lanes 1 to 7 and lanes 8 to 14, respectively), and PCR was performed on these samples with different primer sets. The primers FIGS11 and FIGS12 were used on the samples in lanes 1 to 7 and amplified a 650-bp product. Lanes 8 to 14 represented PCR products obtained using the primers FIGS11 and NPIGS-R, which amplified a 500-bp product from total genomic DNA. The numbers on the left are the molecular weights (Kb) of the Gen-100 bp DNA ladder (GeneMark) (lane M). (B): The macro- and microspores of Fo276 were added into soil particles with serial 10-fold dilutions to generate different spore concentrations ranging from 150,000 to 150 spores/g soil. The primers FIGS11 and NPIGS-R on lane 1 to 5 were able to amplify a 500-bp product from Fo276. The lane corresponded to the following treatments: Lanes 1 to 4 represented soil particles that contained 150,000, 15,000, 1,500 and 150 spores/g soil, respectively. Lane 5 used Fo276 genomic DNA (100 ng/µl) as a positive control. Lane 6 represented sterile dH_2_O added into the soil particles as a negative control.

The results of the PCR sensitivity test showed that the primers FIGS11 and NPIGS-R could amplify the 500-bp fragment from as little as 10 pg (10^-2^ ng) template DNA in a 25-µl reaction mixture (Fig 3). The utility of these primers to detect nonpathogenic *Fo* in roots was shown by the detection of Fo276 in roots of artificially infected cucumber seedlings. A PCR product was obtained with extracted DNA down to a 200-fold dilution. In contrast, with the FIGS11/FIGS12 primer pair, PCR products were only obtained down to an 80-fold dilution. The primers FIGS11/NPIGS-R also detected DNA extracted from soil infested with conidia from the *Fo* isolate Fo276. Using a soil dilution series, the lowest detection limit was 150 conidia/g soil (Fig 4).

### Evaluation of Biocontrol Potential of Nonpathogenic *Fo* Isolates

Seven nonpathogenic *Fo* isolates (Table 1) were evaluated by the pre-inoculation of cucumber seedlings for their potential to delay symptom expression of cucumber Fusarium wilt. Two-week-old seedlings grown in peat moss infested with the nonpathogenic isolates were transplanted into soil infested with the *Foc* isolate Foc100 and observed over an 8-wk period for symptom development. Five of the nonpathogenic *Fo* isolates (Fo95022, Fo95024, Fo95026, Fo276 and Fo366) delayed symptom development. Using the FIGS11/NPIGS-R primer pair, the 500-bp fragment was amplified from the IGS DNA of these five isolates. The other two nonpathogenic *Fo* isolates (Fo95020 and Fo95021) failed to delay symptom development, and no PCR product was amplified by the FIGS11/NPIGS-R primer pair in these two isolates (Table 3). Disease severity was suppressed by the *Fo* isolates Fo95024, Fo95026, Fo276 and Fo366 throughout the 8-wk period following the transplantation of seedlings into *Foc*-infested soil, but isolate Fo95022 suppressed disease severity for only 7 wks. Two *Fo* isolates, Fo276 and Fo95024, were most effective in suppressing disease development; the disease severity ratings they provided after 8 wks were 6% and 14%, respectively, as compared with a 71% rating for the control, which was not pre-inoculated (Table 3). The five isolates, Fo276, Fo366, Fo95022, Fo95024 and Fo95026 that showed efficacy on biological control have been deposited to Bioresource Collection and Research Center (BCRC) in Taiwan with accession no. of FU30079, FU30080, FU30081, FU30082 and FU30083, respectively.

**Table 3:** Efficacy of nonpathogenic *Fusarium oxysporum* isolates to suppress cucumber Fusarium wilt development^a^.

Pre-inoculationtreatment	PCR 500-bp^b^product	Disease severity (%)^c^ after *Fo cucumerinum* inoculation						
			3 wk	4 wk	5 wk	6 wk	7 wk	8 wk
Water (CK)	NA		0	7	25b	43b	57b	71c
95020	-		0	0	57c	39b	57b	71c
95021	-		0	0	14ab	57b	68b	71c
95022	+		0	0	0a	7a	57b	96c
95024	+		0	0	0a	0a	0a	14a
95026	+		0	0	0a	0a	14a	43b
Fo276	+		0	0	0a	6a	6a	6a
Fo366	+		0	10	21b	25b	40b	44b

a The experiment was conducted in a greenhouse (25-35°C) using the substrate infestation inoculation method.

b Plants of each treatment were assayed on a scale of 0-4∶0 =  Healthy plants, 1 =  cotyledon and first leaf with yellowing, 2 =  stunting or <1/2 leaves with yellowing, 3 =  stem yellowing, vascular discoloration, and >1/2 leaves with wilt symptoms, and 4 =  plant wilted or dead. The disease scale was converted to disease severity and rounded off, as described in the Materials and Methods.

c Amplification by primer pair FIGS11/NPIGS-R; NA =  not applicable, - = not.

amplified, + = amplified.

### Use of FIGS11/NPIGS-R to identify *Fo* isolates with biocontrol potential

A total of 77 *Fo* isolates isolated from plant tissues and soil from various locations across Taiwan were assayed by PCR using the FIGS11/NPIGS-R primer pair. The amplification of the 500-bp IGS fragment occurred in only six of the *Fo* isolates, including two from plants (isolates Fo7 and SPA7) and four from soil (isolates HS33, OSS11, OSS12, and OSS14) (Table S1 and Fig S1 with partial gel results). All six isolates identified as PCR positive were found to be nonpathogenic by root dip-inoculation of cucumber seedlings in a greenhouse (Table 4). All six isolates identified as PCR positive and shown to be nonpathogenic were found to suppress Fusarium wilt development (Table 5). The disease severity ratings for the plants pre-infected with nonpathogenic *Fo* isolates ranged from 9-25% 8 wks after being transplanted into *Foc*-infested soil, whereas plants that were not pre-infected received a 63% disease severity rating (Table 5).

**Table 4:** Pathogenicity evaluation of the six *Fo* isolates that were PCR positive using the primers FIGS11 and NPIGS-R.

Isolate	Fungalspecies	Isolationsources	PCR^a^amplification	Pathogenic tocucumber^b^
				
Fo7	*Fusarium oxysporum*	Wax apple	+	-
HS33	*Fusarium oxysporum*	Suppressive Soil	+	-
OSS11	*Fusarium oxysporum*	Rhizosphere Soil	+	-
OSS12	*Fusarium oxysporum*	Rhizosphere Soil	+	-
OSS14	*Fusarium oxysporum*	Rhizosphere Soil	+	-
SPA7	*Fusarium oxysporum*	Sweet potato	+	-
Foc100	*Fusarium oxysporum*	cucumber	-	+
Fol146	*??????????????????*	tomato	-	-

a The symbol “+” means that these isolates could PCR amplify a 500-bp product with the primers FIGS11 and NPIGS-R; all of these products were sequenced and confirmed to have 100% identity with Fo276.

b *F. oxysporum* isolates were tested for their pathogenicity using the root dip assay on cucumber, and the symbol “−” means that there were no symptoms on the cucumber plant 3 weeks after inoculation.

**Table 5:** Biocontrol efficacy of the six *Fusarium oxysporum* isolates shown to be nonpathogenic to cucumber^a^.

Pre-inoculation	Disease severity (%)^b^ after *Fo cucumerinum* inoculation			
treatment	5 wk	6 wk	7 wk	8 wk
Water (CK)^c^	16	27	55a	63a
Fo276	9	11	16b	21b
SPA7	2	4	13b	25b
Fo7	5	5	11b	11b
OSS11	7	13	20b	23b
OSS12	9	11	16b	21b
OSS14	0	0	7b	9b
HS33	3	8	8b	25b

a The experiment was conducted in a greenhouse (18-28°C) using the substrate infestation inoculation method.

b Plants of each treatment were assayed on a scale of 0-4∶0 =  Healthy plants, 1 =  cotyledon and first leaf with yellowing, 2 =  stunting or <1/2 leaves with yellowing, 3 =  stem yellowing, vascular discoloration, and >1/2 leaves with wilt symptoms, and 4 =  plant wilted or dead. The disease scale was converted to disease severity and rounded off, as described in the Materials and Methods.

c The CK treatment was pre-inoculated with distilled water and then transplanted into infested soil with Foc100.

### Phylogenetic Analysis of Nonpathogenic *Fo* Showing Efficacy on Control Cucumber Wilting Based on EF-1α Gene and IGS Region

The DNA products of the EF-1α and IGS region of the five *Fo* isolates (Fo276, Fo366, Fo95022, Fo95024 and Fo95026) amplified by EF-1H/EF-2T and CNS1/CNL12 were 671 bp and 2.4-2.6 kb, respectively. The nucleotide sequences of the EF-1α and IGS region from these five *Fo* isolates were submitted to GenBank with accession numbers as described as follows: Fo276: KC622306/KC622301, Fo366: KC622307/AB683869, Fo95022: KC622308/KC622302, Fo95024: KC622305/KC622303 and Fo95026: KC622309/KC622304.

**Figure 5:**
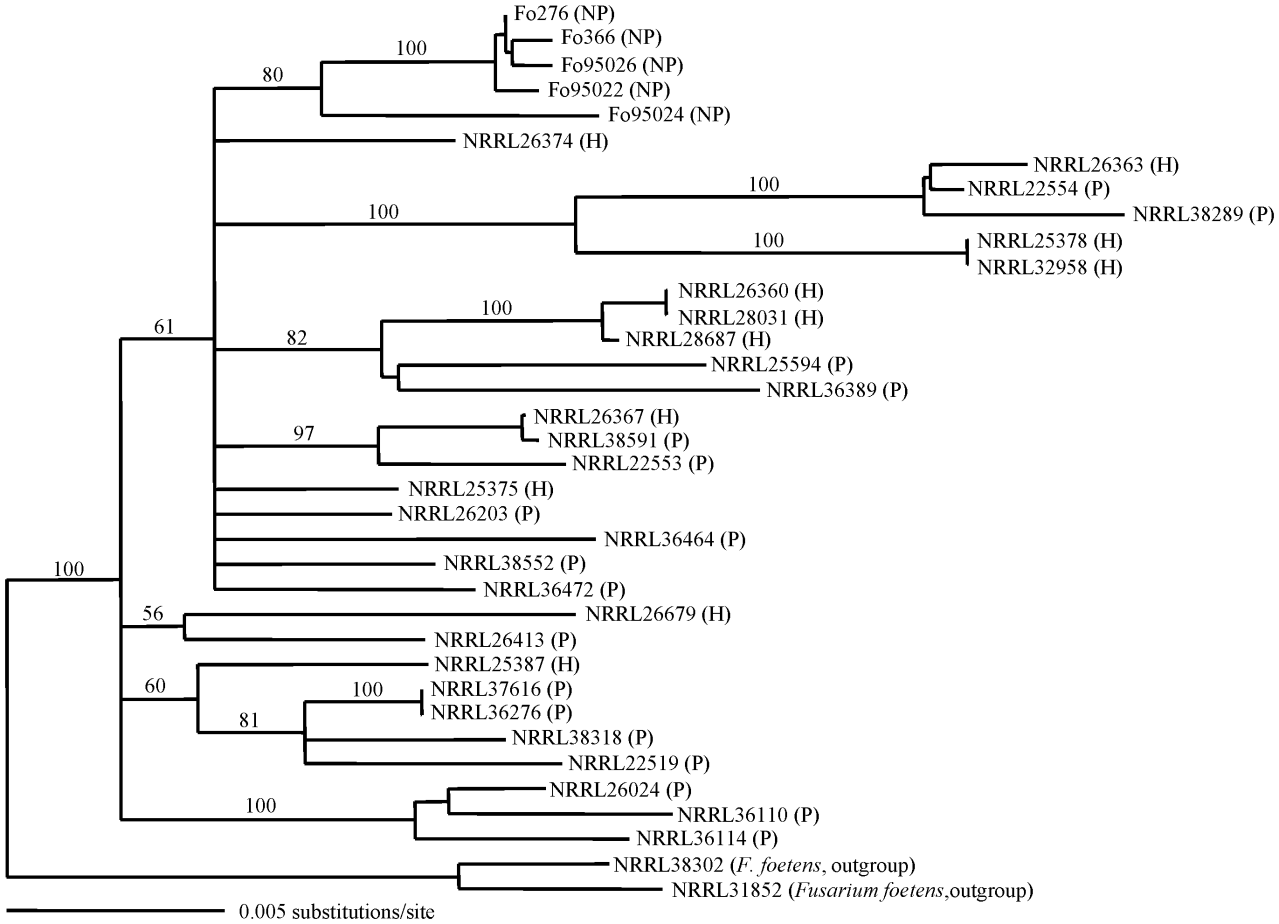
EF-1α and IGS sequence-based tree generated with neighbour-joining analysis. Numbers at branch modes indicate reliable values from bootstrap analysis with 1000 replications. *Fusarium foetens* NRRL38302 and 31852 were used as outgroups to root the tree. NP = nonpathogenic *Fusarium oxysporum* with biological control activity; H = Human pathogen; P = Plant pathogen.

The aligned and truncated EF-1α+IGS sequences consisted of 2,752 characters, with 2374 characters constant, 148 parsimony uninformative and 230 parsimony informative. The NJ tree constructed from the EF-1α+IGS region showed that the five isolates of nonpathogenic *Fo* with biological control activity were fell into one group with 80% bootstrap values and distinct from other pathogenic *Fo* isolates (Fig 5).

## Discussion

Based on the data presented, the new primer NPIGS-R combined with the published general primer FIGS11 [27], [30] distinguishes *Fo* isolates that are both nonpathogenic and potential biocontrol agents for Fusarium wilt of cucumber from formae speciales of *Fo* and other nonpathogenic *Fo* isolates that lack biocontrol potential. However, this primer pair does not distinguish nonpathogenic *Fo* isolates lacking biocontrol potential from formae speciales of *Fo*. Nonpathogenic *Fo* isolates that recovered from Fusarium wilt-suppressive soils have been extensively studied for antagonistic activity against various formae speciales of *Fo* [8]. There are several reports of nonpathogenic *Fo* isolates being used as biological control agents to manage Fusarium wilt of various crops [9], [10], [11], [12], [13]. However, it has been shown that not all nonpathogenic *Fo* isolates possess biocontrol potential [8], [14]. Currently, bioassay is the only available and reliable method to identify *Fo* isolates with biological control potential, but this assay is very time-consuming and laborious. The highly specific FIGS11/NPIGS-R primer set appears to offer an opportunity to rapidly and efficiently screen large numbers of *Fo* isolates to identify those with biocontrol potential.

These new molecular tools were used to investigate the genetic relationships among pathogenicity, biological control and saprophytic *Fo* and to elucidate the genetic determinants of pathogenicity and biological control ability. Appel and Gordon [27] showed an interaction between pathogenic and nonpathogenic *Fo* and addressed the differences in pathogenic race, vegetative compatibility group (VCG), mitochondrial DNA (mDNA) haplotype and IGS haplotype, but could not directly separate nonpathogenic *Fo* from pathogenic *Fo*. Therefore, the genetic basis of pathogenic, nonpathogenic or biocontrol strains of *Fo* remains unclear [39], [40]. In this study, we developed a molecular marker to differentiate the Taiwanese nonpathogenic *Fo* isolates from the pathogenic isolates on cucumbers (Fig 1). The primers FIGS11/NPIGS-R were able to specifically amplify a DNA product from the *Fo* isolates that showed potential for controlling Fusarium wilt of cucumber (Table 1 and Fig 2). The variation in the intergenic spacer (IGS) region of ribosomal DNA is useful for resolving intra-specific relationships within *Fo* [23], [25], [26]. It has been suggested that the variation in the IGS of rDNA may have a considerable effect on development, evolution, and ecology through its effects on growth-rate regulation, resulting from the role of the IGS in the production of rRNA [41]. This study suggests that the variation in the IGS region could differentiate the *Fo* isolates with biological control abilities from the pathogenic and the nonpathogenic Fo isolates that did not have biocontrol potential.

The *Fo* isolates Fo95020 and Fo95021, which showed no PCR-amplified 500-bp product with the primers FIGS11 and NPIGS-R, were unable to delay the disease progression of Fusarium wilt of cucumber in a greenhouse experiment. According to this result, we speculated that these two isolates might belong to one of the *Fo* formae speciales or a saprophytic one that lacks the biological control ability for Fusarium wilt of cucumber. To relieve the concern about the pathogenicity of the nonpathogenic *Fo* used in this study, the *Fo* isolates Fo276 and Fo366 were tested for their pathogenicity on fourteen species of the main cultivated crops or vegetables (such as asparagus bean, basil, bitter gourd, cucumber, loofah, melon, pea, radish, snap bean, spinach, sweet potato, tomato, watermelon and wax gourd) in Taiwan, and the results showed that no symptoms were induced on the inoculated plants by either isolate (Wang, unpublished data). This lack of symptoms may be because the nonpathogenic *Fo* isolates were defined as those that “failed to induce disease on a limited number of plant species to which they had been inoculated” [14]. Therefore, the pathogenicity test may lead to problems in differentiating the isolates. Future research will focus on the utilization of the primers FIGS11 and NPIGS-R to screen more *Fo* isolates with biological control ability and to reveal the difference in genomic or IGS sequence between the pathogenic and nonpathogenic isolates.

The sensitivity of this PCR assay with the primers FIGS11 and NPIGS-R was shown to detect as low as 1×10^-2^ ng of fungal DNA. Such minute amounts of fungal DNA can be obtained easily from several natural substrates or living plant tissues that harbor the target strains. Moreover, 77 isolates of *Fo* from soil and plants tissues were screened and examined with FIGS11 and NPIGS-R. The results obtained demonstrated that only six isolates could be detected by FIGS11 and NPIGS-R. To confirm pathogenicity, the isolates that were amplified by FIGS11 and NPIGS-R had been tested for pathogenicity to cucumber, and no symptoms occurred in plants after inoculation. Meanwhile, these newly selected isolates have shown efficacy in delaying the disease progression of Fusarium wilt of cucumber in a greenhouse experiment. Thus, the newly developed molecular detection method with the primers FIGS11 and NPIGS-R may have practical applications in the study of the epidemiology, fungal population genetics, and even the mechanism of nonpathogenic strains in combating Fusarium wilt diseases [21]. Moreover, as mentioned in the Materials and Methods, the reason why Fo366 lost its biocontrol potential is unknown, but mutation was suspected [42]. This finding reemphasized that additional *Fo* isolates with biological control ability are needed and reaffirms the significance of this new reliable and highly specific protocol in the identification of the biocontrol potential of *Fo* isolates. In addition, several studies on Fusarium disease show that mixtures of biocontrol agents have provided better control and that a range of biocontrol mechanisms may operate in mixed populations of biocontrol agents [8], [43], [44]. Moreover, different biocontrol mechanisms have been shown among different nonpathogenic *Fo* isolates [8] and, the combination of different nonpathogenic *Fo* isolates might use multiple mechanisms to control the Fusarium wilt disease and provide better control efficacy.

Previous studies revealed that the *Fusarium oxysporum* is a species complex (FOSC) containing several groups of isolates with differently pathogenic activity on human and plants (Ma et al., 2010 [45], O’Donnell et al., 2004 [33], O’Donnell et al., 2009 [46]). Our result demonstrated that some nonpathogenic *Fo* isolates of FOSC that showed positive reaction to PCR have activity on controlling Fusarium wilt of cucumber. According to the molecular topology, the *F. oxysporum* with pathogenicity on human or plants were scattered in different molecular groups. The scattered phenomenon of topology in human and plant *Fo* pathogens was also confirmed by O’Donnell et al. (2009 [46]). They also mentioned that the evolutionary relationships between plant pathogens and nonpathogenic *Fo* (untested with the biological control activity) were nested and still unclear (O’Donnell et al., 2009 [46]). However, the isolates of nonpathogenic *Fo* with biological control ability were grouped into a unique clade and differentiated from other reference isolates of human and plant pathogens in this study. Along with these results, the nonpathogenic *Fo* isolates with biological control ability amplified by FIGS11/NPIGS-R were monophylogeny. Thus, the newly designed primer is indeed with the ability of specificity and peculiarity in detecting the nonpathogenic *Fo* with biological control ability in Taiwan.

In the future, additional isolates of different formae speciales and *Fo* nonpathogenic to cucumber from various geographic origins will be used to further confirm the specificity of our PCR assay method for identifying nonpathogenic *Fo* isolates with biocontrol potential. Further studies will determine whether the markers can be used worldwide.

## Supporting Information

Figure S1 **Use of FIGS11/NPIGS-R to identify Fo isolates with biocontrol potential.** Agarose gels showing amplification products of partial isolates of *Fusarium oxysporum* obtained from soils and plant tissues from the field by polymerase chain reaction (PCR). (A): DNA products, 550 to 650 bp in length, were amplified from isolates of *F. oxysporum* by FIGS11 and FIGS12; (B): DNA products, 500 bp in length, were amplified from isolates of *F. oxysporum* by FiGS11 and NPIGS-R. The numbers on the left are the molecular weights (Kb) of the Gen-100 bp DNA ladder (GeneMark) (lane M). Lanes 1 to 17 represented the *Fusarium oxysporum* isolates, which were collected from fields, and lanes 18 to 23 represented the nonpathogenic *Fusarium oxysporum* isolates Fo7, HS33, OSS11, OSS12, OSS14 and SPA7.
(TIFF)

Table S1 **Screening of 77** ***Fusarium oxysporum*** **isolates using PCR with the primers FIGS11 and NPIGS-R.**
(DOC)

## References

[1] Gordon TR, Okamoto D (1990) Colonization of crop residue by *Fusarium oxysporum* f. sp. *melonis* and other species of *Fusarium* . Phytopathology 80: 381–386.

[2] Gordon TR, Okamoto D (1992) Population structure and the relationship between pathogenic and nonpathogenic strains of Fusarium oxysporum. Phytopathology 82: 73–77.

[3] Gordon TR, Okamoto D, Milfroom MG (1992) The structure and interrelationship of fungal populations in native and cultivated soils. Mol Ecol 1: 241–249.

[4] Fourie G, Steenkamp E, Ploetz R, Gordon T, Viljoen A (2011) Current status of the taxonomic position of *Fusarium oxysporum* formae specialis cubense within the *Fusarium oxysporum* complex. Infect Genet Evol 11: 533–542. 21256980 10.1016/j.meegid.2011.01.012

[5] Vakalounkis DJ, Fragkiadakis GA (1999) Genetic diversity of *Fusarium oxysporum* isolates from cucumber: Differentiation by pathogenicity, vegetative compatibility, and RAPD fingerprinting. Phytopathology 89: 161–169. 18944791 10.1094/PHYTO.1999.89.2.161

[6] Erwin DC (1981) Chemical control. In: Marashall EM, Alois AB, Carl HB, editors. Fungal wilt Disease of Plants. New York: Academic Press. 563–594.

[7] Lazarovits G, Tenuta M, Conn KL (2001) Organic amendments as a disease control strategy for soilborne diseases of high-value agricultural crops. Australasian Plant Pathol 30: 111–117.

[8] Fravel D, Olivain C, Alabouvette C (2003) Research review: *Fusarium oxysporum* and its biocontrol. New Phytol 157: 493–502. 10.1046/j.1469-8137.2003.00700.x 33873407

[9] Larkin RP, Fravel DR (1999) Mechanisms of action and dose-response relationships governing biological control of Fusarium wilt of tomato by nonpathogenic *Fusariu*m spp. Phytopathology 89: 1152–1161. 18944639 10.1094/PHYTO.1999.89.12.1152

[10] Ogura K, Komada H (1984) Biological control of Fusarium wilt of sweet potato by nonpathogenic *Fusarium oxysporum* . Ann Phytopathol Soc Japan 50: 1–9.

[11] Lemanceau P, Bakker PA, Kogel WJD, Alabouvette C, Schippers B (1992) Effect of pseudobactin 358 production by *Pseudomonas putida* WCS358 on suppression of fusarium wilt of carnations by nonpathogenic *Fusarium oxysporum* Fo47. Appl Environ Microbiol 58: 2978–2982. 1444411 10.1128/aem.58.9.2978-2982.1992 PMC183036

[12] Chen JF (1999) Identification of Fusarium wilt of cucumber and screening of nonpathogenic *Fusarium oxysporum* for the disease control. Taichung. Taiwan, ROC: National Chung Hsing University. Master’s thesis.

[13] Wang CJ (2006) Study of nonpathogenic *Fusarium oxysporum* in controlling Fusarium wilt of cucumber and asparagus bean. Taichung. Taiwan, ROC: National Chung Hsing University. Master’s thesis.

[14] Alabouvette C, Lemanceau P, Steinberg C (1996) Biological control of Fusarium wilts: opportunities for developing a commercial product. In: Hall R, editor. Managing Soilborne Plant Pathogens. Minnesota: APS Press. 192–212.

[15] Deacon JW (2006) Fungal genetics, molecular genetics, and genomics. In: Deacon JW, editor. Fungal biology. New York: Blackwell Press. 158–183.

[16] Jurado M, Vázquez C, Marín S, Sanchis V, González-Jaéna MT (2006) PCR-based strategy to detect contamination with mycotoxigenic *Fusarium* species in maize. Syst Appl Microbiol 29: 681–689. 16513314 10.1016/j.syapm.2006.01.014

[17] Schilling AG, Möller EM, Geiger HH (1996) Polymerase chain reaction-based assays for species-specific detection of *Fusarium culmorum*, *F. graminearum*, and *F. avenaceum* . Phytopathology 86: 515–523.

[18] Wilson A, Simpson D, Chandler E, Jennings P, Nicholson P (2004) Development of PCR assays for the detection and differentiation of *Fusarium sporotrichioides* and *Fusarium langsethiae* . FEMS Microbiol Lett 233: 69–76. 15043871 10.1016/j.femsle.2004.01.040

[19] Yergeau E, Filion M, Vujanovic V, St-Arnaud M (2005) A PCR-denaturing gradient gel electrophoresis approach to assess Fusarium diversity in asparagus. J Microbiol Methods 60: 143–154. 15590089 10.1016/j.mimet.2004.09.006

[20] Lievens B, Rep M, Thomma BPHJ (2008) Recent developments in the molecular discrimination of formae speciales of *Fusarium oxysporum* . Pest Manag Sci 64: 781–788. 18335459 10.1002/ps.1564

[21] Lin YH, Chang JY, Liu ET, Chao CP, Huang JW, et al (2009) Development of molecular marker for specific detection of *Fusarium oxysporum* f. sp. *cubense* race 4. Eur J Plant Pathol 123: 353–365.

[22] Dissanayake MLMC, Kashima R, Tanaka S, Ito S (2009) Pathogenic variation and molecular characterization of Fusarium species isolated from wilted Welsh onion in Japan. J Gen Plant Pathol 75: 37–45.

[23] Edel-Hermann V, Gautheron N, Steinberg C (2012) Genetic diversity of *Fusarium oxysporum* and related species pathogenic on tomato in Algeria and other Mediterranean countries. Plant Pathol 61: 787–800.

[24] Kawabe M, Katsube K, Yoshida T, Arie T, Tsuchiya K (2007) Genetic diversity of Fusarium oxysporum f. sp. spinaciae in Japan based on phylogenetic analyses of rDNA-IGS and MAT1 sequences. J Gen Plant Pathol 73: 353–359.

[25] Lori G, Edel-Hermann V, Gautheron N, Alabouvette C (2004) Genetic diversity of pathogenic and nonpathogenic populations of *Fusarium oxysporum* isolated from carnation fields in Argentina. Phytopathology 94: 661–668. 18943491 10.1094/PHYTO.2004.94.6.661

[26] Srinivasan K, Spadaro D, Poli A, Gilardi G, Gullino ML, et al (2012) Genetic diversity and pathogenicity of *Fusarium oxysporum* isolated from wilted rocket plants in Italy. Phytoparasitica 40: 157–170.

[27] Appel DJ, Gordon TR (1996) Relationships among pathogenic and nonpathogenic isolates of *Fusarium oxysporum* based on the partial sequence of the intergenic spacer region of the ribosomal DNA. Mol Plant-Microbe Interact 9: 125–138. 8820752 10.1094/mpmi-9-0125

[28] Nash SM, Snyder WC (1962) Quantitative estimation by plate counts of propagules of the bean root rot Fusarium in field soils. Phytopathology 52: 567–572.

[29] Saitoh K, Togashi K, Arie T, Teraoka T (2006) A simple method for a mini-preparation of fungal DNA. J Gen Plant Pathol 72: 348–350.

[30] Kawabe M, Kobayashi Y, Okada G, Yamaguchi I, Teraoka T, et al (2005) Three evolutionary lineages of tomato wilt pathogen, *Fusarium oxysporum* f. sp. *lycopersici*, based on sequences of IGS, MAT1, and pg1, are each composed of isolates of a single mating type and a single or closely related vegetative compatibility group. J Gen Plant Pathol 71: 263–272.

[31] Scher FM, Baker R (1982) Effect of *Pseudomonas putida* and a synthetic iron chelator on induction of soil suppressiveness to Fusarium wilt pathogens. Phytopathology 72: 1567–1573.

[32] O’Donnell K, Sutton DA, Rinaldi MG, Magnon KC, Cox PA, et al (2004) Genetic diversity of human pathogenic members of the *Fusarium oxysporum* complex inferred from multilocus DNA sequence data and amplified fragment length polymorphism analyses: evidence for the recent dispersion of a geographically widespread clonal lineage and nosocomial origin. J Clin Microbiol 42: 5109–5120. 15528703 10.1128/JCM.42.11.5109-5120.2004 PMC525153

[33] Ortoneda M, Guarro J, Madrid MP, Caracuel Z, Roncero MIG, et al (2004) *Fusarium oxysporum* as a multihost model for the genetic dissection of fungal virulence in plants and mammals. Infect Immunity 72: 1760–1766. 14977985 10.1128/IAI.72.3.1760-1766.2004 PMC356063

[34] Mbofung GY, Hing SG, Pryor BM (2007) Phylogeny of *Fusarium oxysporum* f. sp. *lactucae* Inferred from Mitochondrial Small Subunit, Elongation Factor 1-alpha, and Nuclear Ribosomal Intergenic Spacer Sequence Data. Phytopathology 97: 87–98. 18942941 10.1094/PHYTO-97-0087

[35] Thompson JD, Gibson TJ, Plewniak F, Jeanmougin F, Higgins DG (1997) The Clustal X windows interface: flexible strategies for multiple sequence alignment aided by quality analysis tools. Nucleic Acids Res 25: 4876–4882. 9396791 10.1093/nar/25.24.4876 PMC147148

[36] Rambaut A (2000) Se-Al: Sequence Alignment Editor. Oxford, UK: Department of Zoology, University of Oxford.

[37] Kimura M (1980) A simple method for estimating evolutionary rate of base substitutions through comparative studies of nucleotide sequences. J Mol Evol 16: 111–120. 7463489 10.1007/BF01731581

[38] Felsenstein J (1985) Confidence limits on phylogenies: an approach using the bootstrap. Evolution 39: 783–791. 28561359 10.1111/j.1558-5646.1985.tb00420.x

[39] Appel DJ, Gordon TR (1995) Intraspecific variation within populations of *Fusarium oxysporum* based on RFLP analysis of the intergenic spacer (IGS) region of the rDNA. Exp Mycol 19: 120–128. 7614373 10.1006/emyc.1995.1014

[40] Recorbet G, Steinberg C, Olivain C, Edel V, Trouvelot S, et al (2003) Wanted: pathogenesis-related marker molecules for *Fusarium oxysporum* . New Phytol 159: 73–92. 10.1046/j.1469-8137.2003.00795.x 33873682

[41] Gorokhova E, Dowling TE, Weider LJ, Crease TJ, Elser JJ (2002) Functional and ecological significance of rDNA intergenic spacer variation in a clonal organism under divergent selection for production rate. Proc Biol Sci 269: 2373–2379. 12495506 10.1098/rspb.2002.2145 PMC1691159

[42] Zhang Z, Yuen GY, Sarath G, Penheiter AR (2001) Chitinases from the plant disease biocontrol agent, *Stenotrophomonas maltophilia* C3. Phytopathology 91: 204–211. 18944395 10.1094/PHYTO.2001.91.2.204

[43] Xu XM, Jeffries P, Paurasso M, Jeger MJ (2011) Combined Use of Biocontrol Agents to Manage Plant Diseases in Theory and Practive. Phytopathology 101: 1024–1031. 21554184 10.1094/PHYTO-08-10-0216

[44] Guetsky R, Shtienberg D. Elad Y, Fischer E, Dinoor A (2002) Improving biological control by combining biocontrol agents each with several mechanisms of disease suppression. Phytopathology 92: 976–985. 18944023 10.1094/PHYTO.2002.92.9.976

[45] Ma LJ, van der Does HC, Borkovich KA, Coleman JJ, Daboussi MJ, et al (2010) Comparative genomics reveals mobile pathogenicity chromosomes in *Fusarium* . Nature 464: 367–373. 20237561 10.1038/nature08850 PMC3048781

[46] O’Donnell K, Gueidan C, Sink S, Johnston PR, Crous PW, et al (2009) A two-locus DNA sequence database for typing plant and human pathogens within the *Fusarium oxysporum* species complex. Fungal Genet Biol 46: 936–948. 19715767 10.1016/j.fgb.2009.08.006

