# Correction: Modified Primers for the Identification of Nonpathogenic *Fusarium oxysporum* Isolates That Have Biological Control Potential against Fusarium Wilt of Cucumber in Taiwan

**DOI:** 10.1371/annotation/299dd843-75a6-46a0-9e90-70bdb3ff6ede

**Published:** 2014-01-02

**Authors:** Chaojen Wang, Yisheng Lin, Yinghong Lin, Wenhsin Chung

In the last row of Table 4, in the column labeled 'Fungal Species', question marks appear in place of *Fusarium oxysporum*. Please see the corrected Table 4 here: http://plosone.org/corrections/pone.00065093.t004.cn.tif

