# Supplementary figures and images for: Modified Primers for the Identification of Nonpathogenic Fusarium oxysporum Isolates That Have Biological Control Potential against Fusarium Wilt of Cucumber in Taiwan
Source: PLoS One. 2013 Jun 7;8(6):e65093. doi: 10.1371/journal.pone.0065093 (PMC3676385; doi:10.1371/journal.pone.0065093)

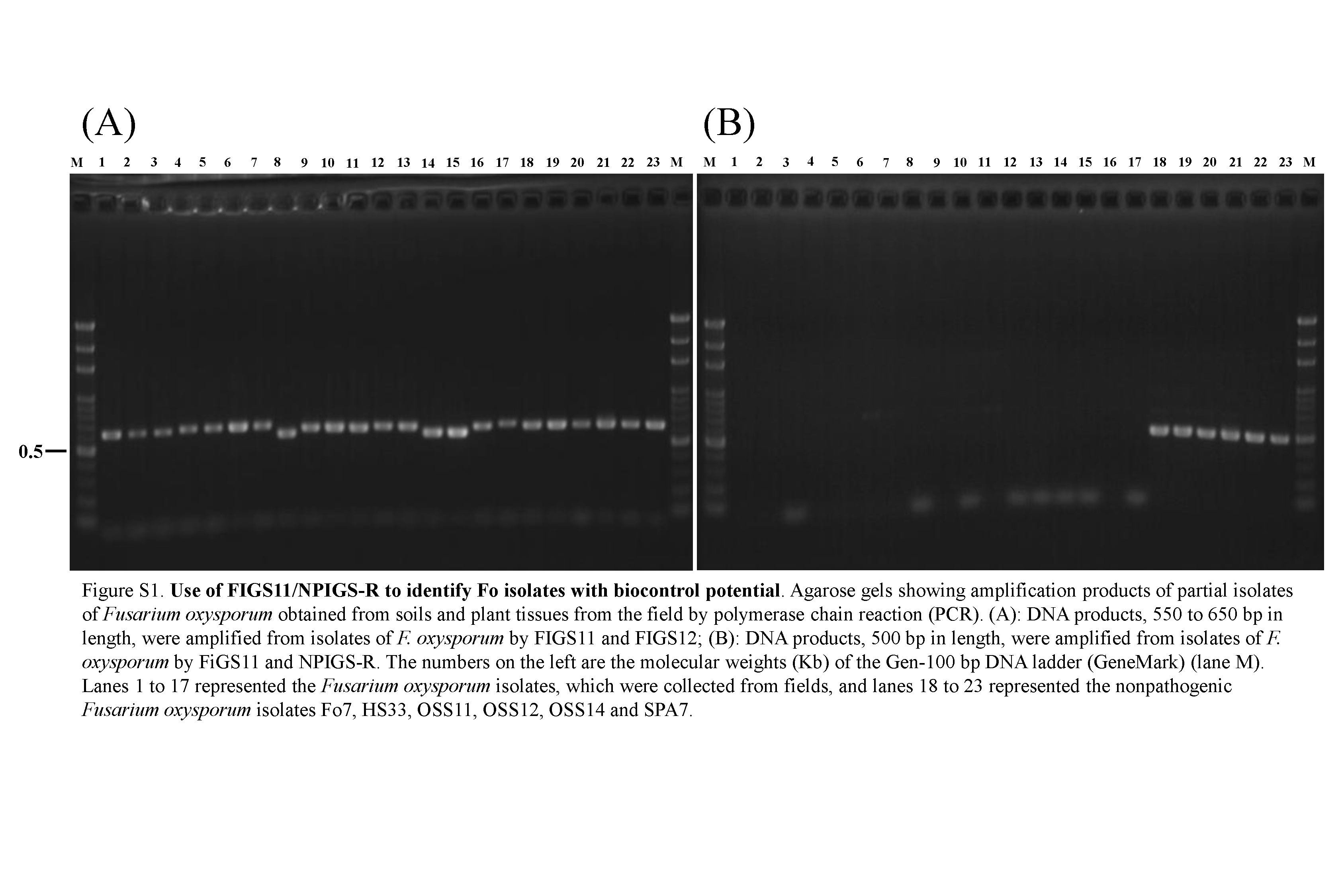

Supplement: Figure S1 — Use of FIGS11/NPIGS-R to identify Fo isolates with biocontrol potential. Agarose gels showing amplification products of partial isolates of Fusarium oxysporum obtained from soils and plant tissues from the field by polymerase chain reaction (PCR). (A): DNA products, 550 to 650 bp in length, were amplified from isolates of F. oxysporum by FIGS11 and FIGS12; (B): DNA products, 500 bp in length, were amplified from isolates of F. oxysporum by FiGS11 and NPIGS-R. The numbers on the left are the molecular weights (Kb) of the Gen-100 bp DNA ladder (GeneMark) (lane M). Lanes 1 to 17 represented the Fusarium oxysporum isolates, which were collected from fields, and lanes 18 to 23 represented the nonpathogenic Fusarium oxysporum isolates Fo7, HS33, OSS11, OSS12, OSS14 and SPA7. (TIFF) [file pone.0065093.s001.tif]
