# Supplementary material for: Modified Primers for the Identification of Nonpathogenic Fusarium oxysporum Isolates That Have Biological Control Potential against Fusarium Wilt of Cucumber in Taiwan
Source: PLoS One. 2013 Jun 7;8(6):e65093. doi: 10.1371/journal.pone.0065093 (PMC3676385; doi:10.1371/journal.pone.0065093)
Supplement: Table S1 — Screening of 77 Fusarium oxysporum isolates using PCR with the primers FIGS11 and NPIGS-R. (DOC) [file pone.0065093.s002.doc]

Table S1. Screening of 77 *Fusarium oxysporum* isolates using PCR with the primers FIGS11 and NPIGS-R.

| Source | Location | Number of isolates | PCR amplification^a^ | |
| --- | --- | --- | --- | --- |
|  |  |  | Yes | No |
| Soil | Pingtung | 19 | 3 | 16 |
|  | Hualien | 27 | 1 | 26 |
|  | Gaushung | 7 | 0 | 7 |
|  | Chiayi | 2 | 0 | 2 |
|  | Nantou | 2 | 0 | 2 |
|  | Taichung | 6 | 0 | 6 |
| Plant | Taipei | 3 | 1 | 2 |
|  | Chiayi | 7 | 1 | 6 |
|  | Nantou | 4 | 0 | 4 |

^a^ These isolates were subjected to amplification with the modified primers FIGS11/NPIGS-R by PCR. Yes means positive for amplification and that it could amplify a 500-bp product, No means negative for amplification. The numerical character indicates the PCR detection results of the amount of *F. oxysporum* isolates collected from the fields.
